# The unusual predominance of maintenance DNA methylation in *Spirodela polyrhiza*

**DOI:** 10.1093/g3journal/jkae004

**Published:** 2024-01-08

**Authors:** Alex Harkess, Adam J Bewick, Zefu Lu, Paul Fourounjian, Todd P Michael, Robert J Schmitz, Blake C Meyers

**Affiliations:** Donald Danforth Plant Science Center, St Louis, MO 63132, USA; Department of Genetics, University of Georgia, Athens, GA 30602, USA; Department of Genetics, University of Georgia, Athens, GA 30602, USA; Waksman Institute of Microbiology, Rutgers University, New Brunswick, NJ 08901, USA; Salk Institute for Biological Studies, La Jolla, CA 92037, USA; Department of Genetics, University of Georgia, Athens, GA 30602, USA; Donald Danforth Plant Science Center, St Louis, MO 63132, USA; Division of Plant Sciences, University of Missouri—Columbia, Columbia, MO 65211, USA

**Keywords:** duckweed, RdDM, methylation, 5mC, H3K9me2

## Abstract

Duckweeds are among the fastest reproducing plants, able to clonally divide at exponential rates. However, the genetic and epigenetic impact of clonality on plant genomes is poorly understood. 5-methylcytosine (5mC) is a modified base often described as necessary for the proper regulation of certain genes and transposons and for the maintenance of genome integrity in plants. However, the extent of this dogma is limited by the current phylogenetic sampling of land plant species diversity. Here we analyzed DNA methylomes, small RNAs, mRNA-seq, and H3K9me2 histone modification for *Spirodela polyrhiza*. *S. polyrhiza* has lost highly conserved genes involved in de novo methylation of DNA at sites often associated with repetitive DNA, and within genes, however, symmetrical DNA methylation and heterochromatin are maintained during cell division at certain transposons and repeats. Consequently, small RNAs that normally guide methylation to silence repetitive DNA like retrotransposons are diminished. Despite the loss of a highly conserved methylation pathway, and the reduction of small RNAs that normally target repetitive DNA, transposons have not proliferated in the genome, perhaps due in part to the rapid, clonal growth lifestyle of duckweeds.

## Teaser

Duckweeds are among the fastest reproducing plants on the planet. One species, Spirodela polyrhiza, has lost key genes involved in *de novo* methylation of DNA at sites often associated with repetitive DNA, and within genes. However symmetrical DNA methylation and heterochromatin is maintained.

## Introduction

**Fig. 1.**
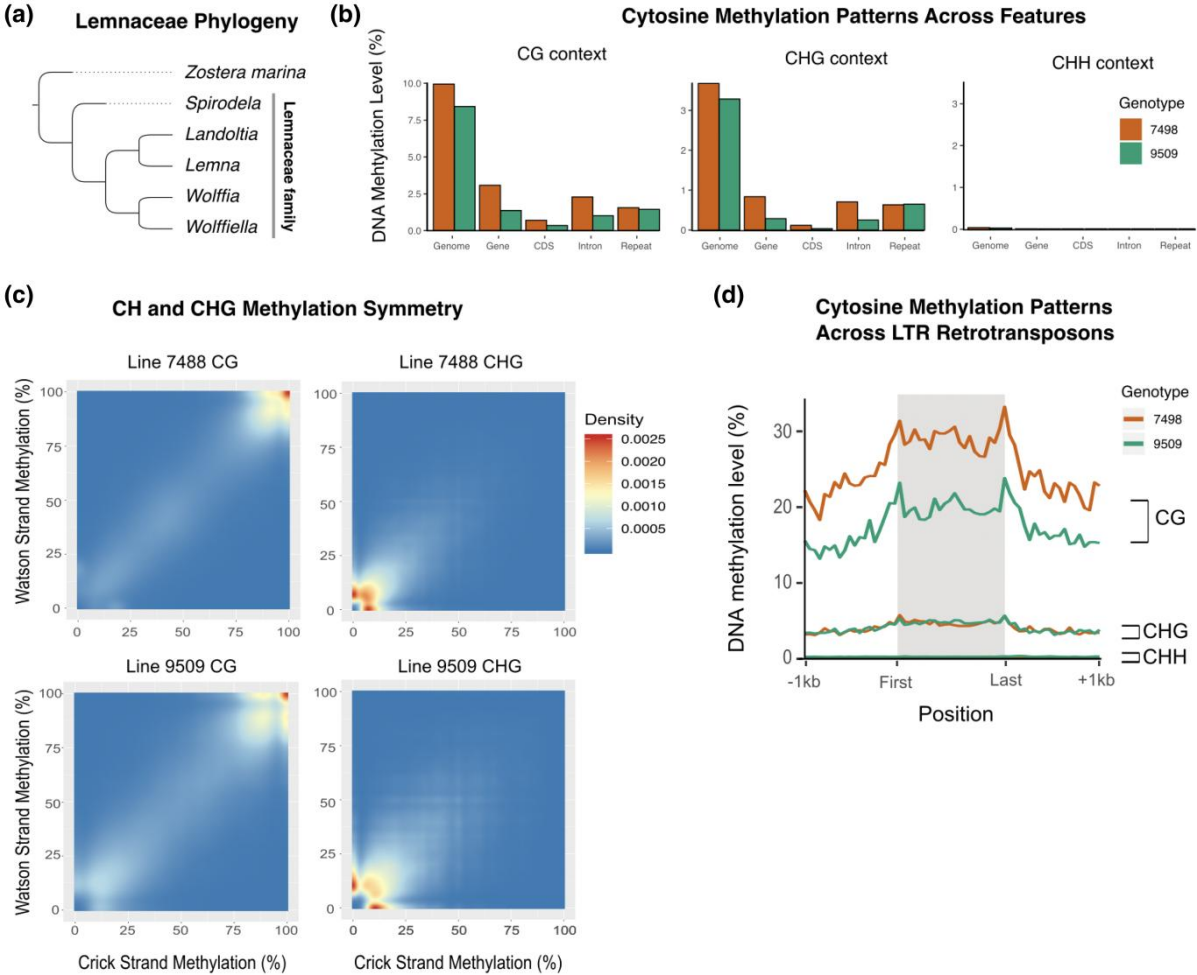
a) A condensed species tree of the Lemnaceae family, with 5 genera: *Spirodela, Landoltia, Lemna, Wolffia,* and *Wolffiella.* b) DNA methylation level for whole genome, genes, coding sequence (CDS), introns, and repeats, across CG, CHG, and CHH site contexts, in 2 genotypes of *S. polyrhiza*. c) Sitewise methylation symmetry of CG and CHG on Watson and Crick strands. d) DNA methylation levels across LTR retrotransposons, across CG, CHG, and CHH sites, in 2 genotypes of *S. polyrhiza*.

Evolutionary theory predicts that asexual populations should become less fit over time due to an irreversible accumulation of deleterious alleles (Lynch *et al*. 1993). Duckweeds are perhaps the most striking counter-example in plants, given their cosmopolitan distribution and ability to survive in diversely harsh environments (Crawford *et al*. 2006). Duckweed is a common name for all 36 species in the Lemnaceae family of monocots, divided across 5 genera: *Spirodela, Lemna, Landoltia, Wolffia,* and *Wolffiella* (Fig. 1a). Most duckweed species rarely flower, instead reproducing primarily by rapid, clonal reproduction that occurs at one of the fastest rates in any angiosperm (Wang *et al*. 2014). The physically largest duckweed species *Spirodela polyrhiza* (∼1 cm wide) intriguingly has the smallest genome size (∼158 megabases) (Wang *et al*. 2014; Michael *et al*. 2017; Harkess *et al*. 2021), and several genome assemblies consistently annotate fewer than 20,000 genes (Wang *et al*. 2014; An *et al*. 2019; Harkess *et al*. 2021). Compared to the *Arabidopsis thaliana* genome which is roughly the same total genome size, *S. polyrhiza* has nearly 25% fewer genes. Without much meiotic recombination through sexual reproduction, and fewer genes for selection to act upon, epigenetic variation could instead be a promising mechanism to explain the global success of clonal duckweeds (Dodd and Douhovnikoff 2016; Shahryary *et al*. 2020).

Sexual reproduction in plants is often accompanied by widespread genome-wide reinforcement of DNA methylation with localized epigenetic reprograming in gametes (Slotkin *et al*. 2009; Schoft *et al*. 2011; Calarco *et al*. 2012; Ibarra *et al*. 2012; Park *et al*. 2017). This results in patterns of both stable DNA methylation inheritance and infrequent spontaneous epialleles (Hofmeister *et al*. 2017). However, nearly 60% of global crops can be bred through clonal propagation (Meyer *et al*. 2012), highlighting the need to illustrate how epigenetics can be used to improve plant breeding efforts. Cytosine DNA methylation or 5-methylcytosine (5mC), is found in species spanning the flowering plant phylogeny (Niederhuth *et al*. 2016). As the number and phylogenetic diversity of plant genomes and DNA methylomes increases, so does the observed diversity of 5mC levels, specificity and DNA methyltransferase enzymes. 5mC DNA methylation in plants occurs in 3 major sequence contexts, each of which requires different sets of enzymes to function: CG, CHG, and CHH (where H = A, C, T). Methylation in these different contexts is established by both de novo and maintenance methyltransferase enzymes. DNA methylation at CG and CHG sites is typically symmetrical across the Watson and Crick strands, whereas DNA methylation at CHH sites is asymmetrical. The observed symmetry is due to the mechanisms by which 5mC is maintained after DNA replication. Methylation at CG sites relies on the maintenance methyltransferase METHYLTRANSFERASE 1 (MET1) (Finnegan *et al*. 1998; Cokus *et al*. 2008; Lister *et al*. 2008), whereas maintenance of methylation at CHG sites relies on a positive feedback loop between dimethylation of lysine 9 on histone 3 (H3K9me2) and CHROMOMETHYLASE 3 (CMT3) (Lindroth *et al*. 2001; Jackson *et al*. 2002; Du *et al*. 2012, 2015). DNA methylation at CHH sites is asymmetrical and is further classified into CWA (where W = A or T) and non-CWA, based on targeting by CMT2 or by 24-nt siRNAs and DOMAINS REARRANGED METHYLTRANSFERASE 2 (DRM2), which are associated with the RNA-directed DNA methylation (RdDM) pathway, respectively (Gouil and Baulcombe 2016; Erdmann and Picard 2020).

Variation in DNA methylation has been connected to pathogen response (Dowen *et al*. 2012), temperature tolerance (Shen *et al*. 2014), and geography (Kawakatsu *et al*. 2016), which could be crucial attributes for clonal duckweeds given their reduced ability to maintain genetic variation through recombination. Several studies have identified unique features of some duckweed species. For instance, the duckweed *S. polyrhiza* displays particularly low levels of 5mC, with evidence that low DNA methylation levels are likely related to its small genome size with the low amounts of repetitive DNA (Michael *et al*. 2017). However, the mechanisms underlying this variation in DNA methylation are unknown (An *et al*. 2019). Immunostaining using antibodies against H3K9me2 suggests that heterochromatin in duckweeds is dispersed rather than concentrated at centromeric and pericentromeric regions (Cao *et al*. 2015). Some lines of *S. polyrhiza* show an abnormal distribution of small RNAs, including a relatively low frequency of 24 nucleotide small RNAs (Fourounjian *et al*. 2019) that might be connected to reduced expression of some RdDM components including *DICER-LIKE 3* (DCL3) (An *et al*. 2019).

Here we further dissect 5mC DNA methylation patterns, histone modifications, small RNAs, and the genes that control major methylation and RdDM pathways in *S. polyrhiza*. We connect observations from the literature and generate additional data to show that *S. polyrhiza* has lost the activity of canonical DNA methylation and small RNA pathway genes that consequently diminish gene body methylation, the RNA-directed DNA methylation pathway, and genome-wide CHH methylation. For instance, previous studies have described genome-wide CHH methylation in *S. polyrhiza* as being low, and that gene bodies lack CG methylation (Michael *et al*. 2017). Here we discover that CHH methylation is entirely absent due to the loss of CMT2 in *S. polyrhiza*. However, maintenance methylation at CG loci is normal. Whereas it was previously reported using antibody immunostaining that *S. polyrhiza* lacked localized patterns of heterochromatin (Cao *et al*. 2015), we instead find clear pericentromeric H3K9me2 peaks on most chromosomes using chromatin immunoprecipitation sequencing (ChIP-seq). Our observations and data provide a new perspective on the interplay between DNA methylation, RdDM and chromatin in *S. polyrhiza*, and build hypotheses about the necessity of these mechanisms in clonal plants.

## Materials and methods

### DNA methylation sequencing and sequence alignment

For all analyses in this study, sterile plants were clonally grown in 0.5X Shenk and Hildebrandt salts at 16-hour days. Whole-genome bisulfite sequencing data for *S. polyrhiza* 7498 and 9509 were generated according to (Urich *et al*. 2015) using whole plants as input. Single-end short-read libraries (150 bp) were aligned using the methylpy pipeline (Schultz *et al*. 2016) to the *S. polyrhiza* 7498 and 9509 genomes. Methylpy calls programs for read processing and aligning: (1) reads were trimmed of sequencing adapters using Cutadapt (Martin 2011), (2) and then mapped to both a converted forward strand (cytosines to thymines) and converted reverse strand (guanines to adenines) using bowtie (Langmead *et al*. 2009). Reads that mapped to multiple locations, and clonal reads were removed. The chloroplast genome (GenBank: JN160603.2) was used to estimate the rate of sodium bisulfite nonconversion.

### DNA methylation analyses

DNA methylation levels were estimated as weighted DNA methylation, which is the total number of aligned DNA methylated reads divided by the total number of methylated plus unmethylated reads with a minimum coverage of at least 5 reads (Schultz *et al*. 2012). Global weighted DNA methylation was estimated across the entire genome, within intergenic regions, transposons, genes (exons + introns), exons, and introns. Additionally, the genome was divided into nonoverlapping 50,000 bp windows, and weighted DNA methylation was estimated for each window.

For metaplots, the locus body (start-to-stop codon for genes and first-to-last bp for transposons) was divided into 20 proportional windows based on locus length. Within gene bodies, only sequenced reads mapping to coding, and exonic DNA were used. Additionally, 1,000 bp upstream and downstream were divided into 20 proportional windows. A single weighted DNA methylation value was calculated for each window across all loci.

For each gene, a binomial test with a Benjamini–Hochberg false discovery rate (FDR) correction was applied to determine the enrichment of DNA methylation at the 3 sequence contexts (CG, CHG, and CHH). Only CG, CHG, and CHH sites found within the coding, exonic sequences were considered. The weighted DNA methylation level of cytosines at CG, CHG, and CHH sites across all coding regions were used as the probability of success, respectively. Enrichment tests for gene body methylation were performed using code from (Zhang *et al*. 2020), found at https://github.com/schmitzlab/Natural_variation_in_DNA_methylation_homeostasis_and_the_emergence_of_epialleles.

To determine per-site methylation levels, the weighted DNA methylation level for each cytosine with ≥3 reads of coverage was calculated. Additionally, DNA methylation levels of symmetrical cytosines (CG or CWG, W = A|T) with ≥3 sequencing coverage were estimated for each strand (Watson and Crick). All plots were generated in R v3.2.4 (https://www.r-project.org/).

### sRNA and mRNA sequencing analysis

sRNA sequencing reads were generated using whole plant total RNA isolated using TRI reagent and the Somagenics RealSeq-AC kit with 100 ng of total RNA as input. Reads were adapter-trimmed with cutadapt v2.0 (Martin 2011) with options “–m 15 TGGAATTCTCGGGTGCCAAGG”. Cleaned reads were aligned to the reference genome using bowtie with settings “-a -v 0” to only report end-to-end alignments with zero mismatches.

Raw mRNA-Seq reads from strain 9509 were retrieved from the Sequence Read Archive (SRR3090696), cleaned with Trimmomatic v0.32 with settings “ILLUMINACLIP:2:30:10 LEADING:3 TRAILING:3 SLIDINGWINDOW:4:15 MINLEN:50” and aligned to the reference genome with TopHat v2.1.1 with default settings other than “-i 25”. Per-gene expression was calculated with Cufflinks v2.2.1 with default settings.

### ChIP-seq and analysis

ChIP was performed as previously described (Zaratiegui *et al*. 2011). Briefly, 1 g of fresh duckweed plantlets were crosslinked in 1% formaldehyde for 10 min. Nuclei were then isolated and sonicated for 15 min, twice. Histone-DNA complexes were pulled down with anti-H3K9me2 (Cell Signaling Technology antibody #9753 s). DNA was isolated and used to prepare ChIP-seq libraries with the TruSeq ChIP Library Preparation Kit (Illumina, IP-202-1012). Sequencing was performed on an Illumina NextSeq500 in the Georgia Genomics and Bioinformatics Core (GGBC) at the University of Georgia.

Raw ChIP reads were trimmed for adapters and low-quality bases using Trimmomatic with the following options: reads were trimmed for TruSeq version 3 single-end adapters with a maximum of 2 seed mismatches, palindrome clip threshold of 30, and simple clip threshold of 10. Trimmed reads were mapped to the genome using bowtie1 with “-v 2 –best –strata -m 1” (Langmead *et al*. 2009). Mapped reads were sorted using SAMtools (Li and Durbin 2009) and then clonal duplicates were removed using Picard (http://broadinstitute.github.io/picard/). The remaining reads were converted to browser extensible data (BED) format with bedtools (Quinlan and Hall 2010). H3K9me2 enriched regions were identified with MACS2 with parameter “–keep-dup all –broad” (Zhang *et al*. 2008). Enrichment of H3K9me2 overlaps with long terminal repeat (LTR) retrotransposons was tested using a Fisher's Exact Test implemented in bedtools v2.26.0.

### Phylogenetic analyses

CMT protein sequences were obtained from (Bewick *et al*. 2017), and additional sequences were identified in monocot species listed on Phytozome v12 (https://phytozome.jgi.doe.gov/pz/portal.html) using best basic local alignment search tool (BLASTP) hit e-value ≤ 1E–06 and bit score ≥ 200 to *A. thaliana* CMT1 (AT1G80740.1), CMT2 (AT4G19020.1), and CMT3 (AT1G69770.1). Similarly, DCL homologs were identified in all monocot species listed on Phytozome v12 using the best BLASTP hit to *A. thaliana* DCL1 (AT1G01040.2), DCL2 (AT3G03300.1), DCL3 (AT3G43920.2), and DCL4 (AT5G20320.1). Protein sequences were aligned using the program PASTA with default parameters. Following alignment, GBblocks were used to identify conserved amino acid positions. All parameters were kept at the default setting except –b2 = *n*0.66 where *n* is the number of sequences and –b5 = h. Bayesian evolutionary analysis by sampling trees (BEAST) v2.3.2 was used to estimate the phylogeny with a BLOSUM62 substitution matrix. The Markov chain Monte Carlo in BEAST was allowed to run until stationarity and convergence (ESS ≥200) was reached, and was assessed using the program Tracer v1.6. A maximum clade credibility tree was generated from the posterior distribution of trees with the burn-in removed using the program TreeAnnotator v2.3.2. Finally, the program FigTree (http://tree.bio.ed.ac.uk/software/figtree/) was used to visualize the tree and exported for stylization. Alignment, site filtering, and tree estimation were performed identically and separately for CMT and DCL phylogenies.

### Comparative transcriptome analyses

To estimate the phylogenetic placement of the loss of DCL2 and DCL3 expression, sequence read archive RNA-seq data were downloaded for *Landoltia punctata* (SRR647050*)* and *Lemna minor* (SRR2917879). Data were cleaned and assembled using Trinity v2.5.1 with default options. Assemblies were subject to blastx searches (1e–10) against the present, but not expressed *Spirodela* DCL3 gene model annotation predicted peptide (Spipo14G0010100).

### LTR retrotransposon annotation

LTR retrotransposons were annotated de novo using GenomeTools LTRharvest with options “-similar 85 -mindistltr 1,000 -maxdistltr 15,000 -mintsd 5 -maxtsd 20”.

## Results

### Maintenance DNA methylation is functional, but CHH methylation is absent

To test if low levels of 5mC might be a conserved feature across the diversity of *S. polyrhiza* (Michael *et al*. 2017), we performed whole-genome bisulfite sequencing across 2 different genotypes (lines 7498 and 9509) (Supplementary Table 1). Both genotypes show similar patterns: roughly 10% of the CG sites in the genome are methylated (Fig. 1b). Fewer than 3.28 and 3.67% of CHG and 0.0065 and 0.035% CHH sites are significantly methylated (Fig. 1b). In *S. polyrhiza,* mCG and some mCHG (specifically CAG and CTG) are symmetrically maintained through equal DNA methylation on the Watson and Crick strands, which are normal features of maintenance methylation (Fig. 1c). However, the maintenance of mCHG in *S. polyrhiza* is weak in comparison to other species that possess a functional CMT3 (Niederhuth *et al*. 2016). CG methylation is present at small clusters of LTR retrotransposons in the genome (Fig. 1d), but CHH methylation which is normally enriched in repetitive elements like LTR retrotransposons (Noshay *et al*. 2019), is absent (Fig. 1c–d).

### A reduction of 24 nucleotide heterochromatic sRNAs

**Fig. 2.**
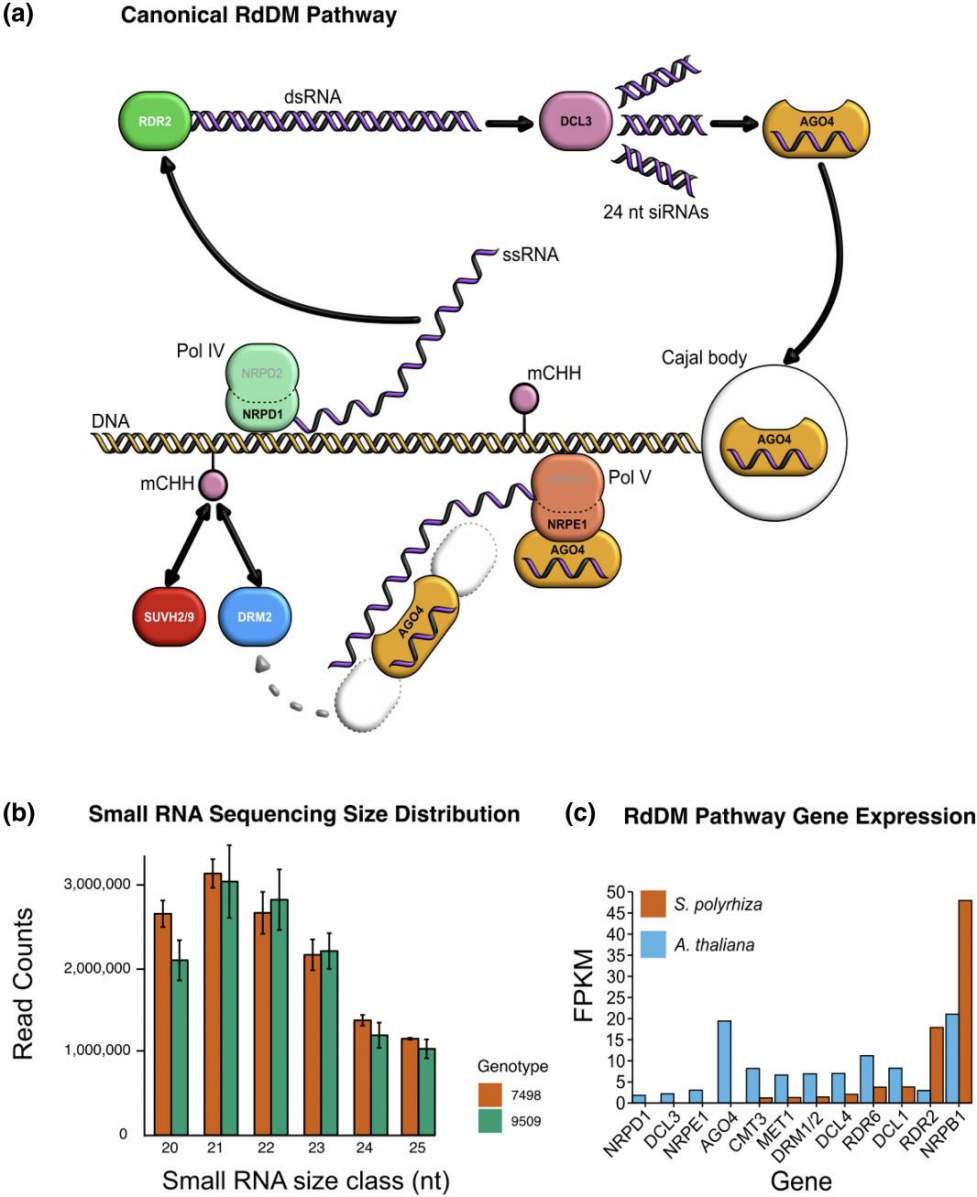
a) Diagram of the canonical RNA-directed DNA methylation (RdDM) pathway in plants. RNA Polymerase IV (Pol IV) transcribes a single-stranded RNA (ssRNA) which is converted to a dsRNA by RNA-DIRECTED RNA POLYMERASE 2 (RDR2). DCL3 then cleaves those dsRNA products into 24 nucleotide small RNA (sRNA) products. One strand of each sRNA is loaded into AGO4, and the AGO-sRNA complex binds to complementary RNA sequences transcribed by RNA Polymerase V (Pol V), guided by interaction with SUVH2 and SUVH9. DRM2 is then recruited, which guides methylation of DNA at those sites. b) The distribution of small RNA sequence read abundance between 20 and 25 nucleotides in 2 genotypes of *S. polyrhiza*. c) Gene expression in *S. polyrhiza* line 9509 and *A. thaliana* measured by RNA-seq for several RdDM and methylation-related genes.

DNA methylation at some CHH sites is guided by small RNAs (sRNAs) generated through the RdDM pathway (Matzke and Mosher 2014) (Fig. 2a), so small RNA (sRNA) sequence reads were generated for both *S. polyrhiza* lines to test for functional defects in the pathway (Supplementary Table 2). Both lines display a distinct lack of 24-nucleotide (nt), heterochromatic siRNAs (het-siRNAs) which are typically the most abundant size class of angiosperm sRNAs (Ma *et al*. 2015; Patel *et al.* 2021) (Fig. 2b). However, Fourounjian *et al*. (2019) previously reported that *S. polyrhiza* line 7498 is dominated by 22 nt sRNAs, with a low level of 21, 23, and 24 nt sRNAs. Our data for both *S. polyrhiza* lines 7498 and 9509 show a different pattern where 21 nt miRNAs are the dominant class, and 24 nt sRNAs are very lowly expressed (Fig. 2b). This could be attributed to the use of ABI SOLiD sRNA sequencing in earlier work (Fourounjian *et al*. 2019) compared to Illumina sRNA sequencing here, which differ in their adapter attachment strategy (ligation in Illumina protocols vs hybridization in SOLiD) and leads to variation in sRNA sequence lengths and composition (Tian *et al*. 2010).

### A variety of RdDM, methylation, and small RNA pathway genes are not expressed

The highly conserved, canonical RdDM pathway produces these 24 nt het-siRNAs via DCL3 processing of an RNA Polymerase IV (Pol IV)-derived double-stranded RNA (dsRNA) (Zhang *et al*. 2007; Mosher *et al*. 2008). These DCL3-derived sRNAs are loaded into ARGONAUTE 4 (AGO4) and guided to their sites of action (Fig. 2a) (Zilberman *et al*. 2003; Li *et al*. 2006; Matzke and Mosher 2014). Due to the reduction of 24 nt het-siRNAs, available whole plant mRNA-seq data was mined for evidence of the expression of RdDM-related genes (Fig. 2c). *DCL3* is present in the genome as a seemingly full-length sequence with no in-frame stop codons, but no gene expression was detectable (FPKM <1), as was also found in another study (An *et al*. 2019). *DCL3* expression is also not detected under various growth and stress conditions in *S. polyrhiza*, including copper, kinetin, nitrate and sucrose additions (Fourounjian *et al*. 2019) (Supplementary Fig. 1). The *DCL3* upstream region is short (fewer than 200 nt), and possibly interrupted by another gene, which may entirely disrupt *DCL3* gene activity (Supplementary Fig. 2).

Given an absence of detectable *DCL3* expression (Fig. 2c), we investigated the presence and expression of orthologs of other plant Dicer-like genes (*DCL1*, *DCL2*, *DCL4*). *DCL1*, which functions in microRNA (miRNA) production, is expressed and produces many conserved miRNAs, indicating it functions normally (Hoang *et al*. 2018). However, *DCL2*, which functions largely in viral defence (Parent *et al*. 2015), is missing from the *S. polyrhiza* genome (Supplementary Fig. 3). *DCL4*, which generates 21-nt siRNAs, is present in the genome and expressed (Gasciolli *et al*. 2005; Xie *et al*. 2005; Yoshikawa *et al*. 2005). *DCL5*, which is implicated in phased siRNA production in maize (Fei *et al*. 2013) and has a role in flower fertility (Teng *et al.* 2020), is also not present in the genome (Patel *et al.* 2021) (Supplementary Fig. 3). In addition to *DCL2* and *DCL3*, there was no detectable expression for *AGO4*, nor the genes encoding the 2 major catalytic subunit genes of the Pol IV complex (*NRPD1* and *NRPE1*) that transcribe single-stranded RNA precursors from RdDM regions and are required for siRNA and methylation-dependent heterochromatin formation (Onodera *et al*. 2005) (Fig. 2c). *CMT3* and *MET1* are expressed in *S. polyrhiza*, consistent with their roles in the maintenance of CG and CHG methylation in the *A. thaliana* genome (Fig. 2c).

We next tested whether the lack of expression of some RdDM genes is a conserved phenomenon across some or all duckweed species in the Lemnaceae family. *De novo* transcriptome assemblies of publicly available whole plant RNA-seq data for species from 2 genera of duckweeds, *Landoltia punctata* and *Lemna minor* (Fig. 1a), were interrogated for *DICER-LIKE* gene family expression. In both *L. punctata* and *L. minor*, de novo transcripts were assembled for *DCL1* and *DCL4*, however, there were no assemblies with BLASTX hits (1e–10) to *DCL2* and *DCL3*. Although whole-genome assemblies of species representing at least all 5 genera of duckweeds will be needed to definitively test this hypothesis, these data suggest that the expression loss of *DCL2* and *DCL3*, possibly leading to the loss of canonical RdDM, may be a widespread phenomenon across several genera of the Lemnaceae family (Fig. 1a).

### The loss of CMT2 and CHH methylation

**Fig. 3.**
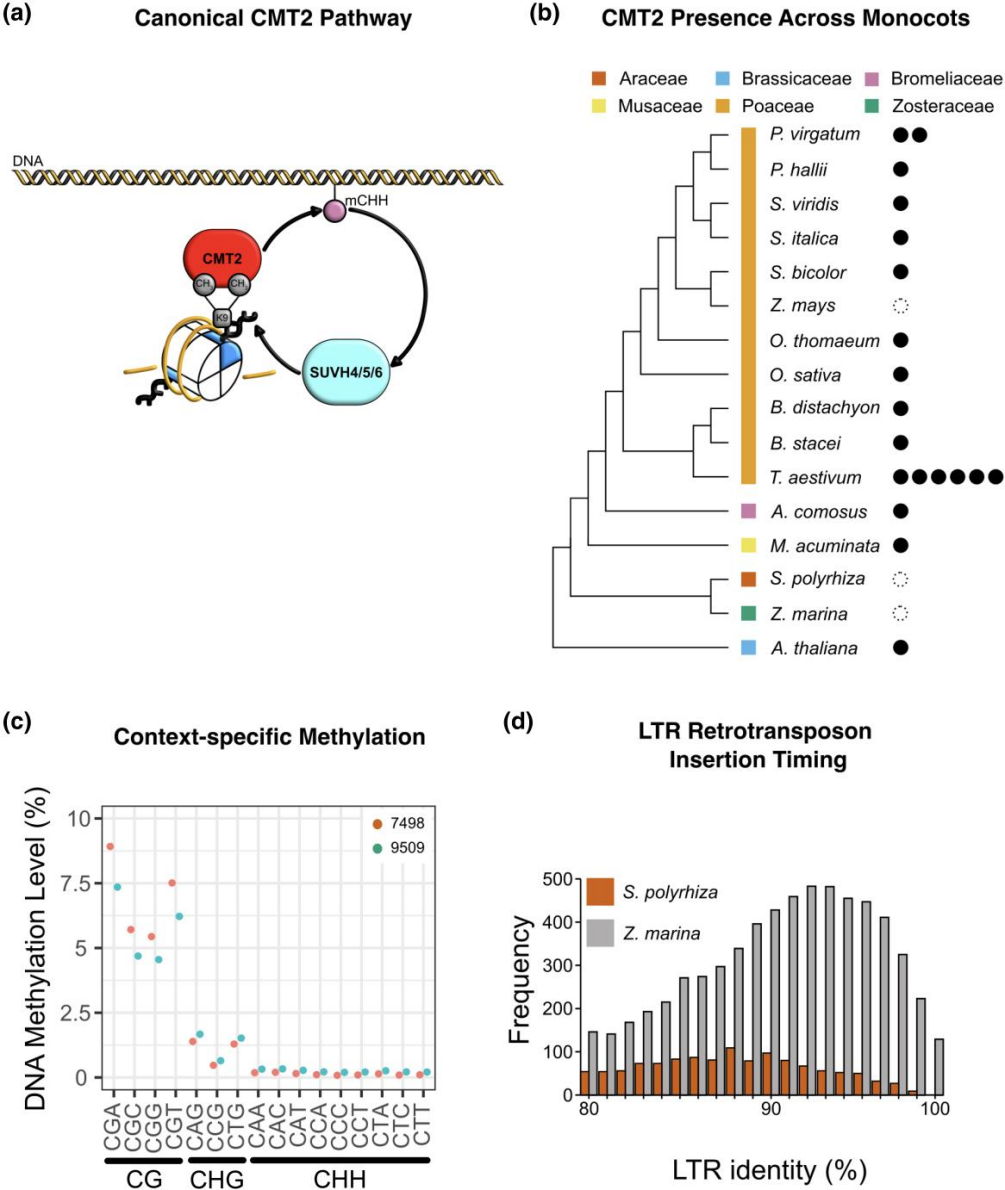
a) Diagram of the canonical CMT2 pathway. b) Presence (filled circles) and absence (empty circles) of CMT2 homologs in genomes across the monocots. c) Genome-wide DNA methylation of 2 genotypes of *S. polyrhiza* split into all possible contexts. d) Relative LTR retrotransposon insertion timings between *S. polyrhiza* and *Z. marina*, based on LTR percent identity comparisons.

Although RdDM is 1 route to forming CHH methylation, an RdDM-independent mechanism is through the action of CMT2, a plant-specific DNA methyltransferase that is highly conserved across angiosperms (Stroud *et al*. 2014; Gouil and Baulcombe 2016; Bewick *et al*. 2017) (Fig. 3a). CHH sites targeted by the RdDM pathway typically show enrichment in all contexts (Gouil and Baulcombe 2016; Wendte *et al*. 2019), which *S. polyrhiza* does not exhibit (Fig. 3a). In *A. thaliana,* CHH methylation deposited via CMT2 can be distinguished from RdDM-targeted sites given that they show an enrichment of CWA methylation (W = A or T) relative to other contexts (Gouil and Baulcombe 2016; Wendte *et al*. 2019) and they are enriched at regions possessing H3K9me2 (Stroud *et al*. 2014). However, a *CMT2* homolog is missing from the *S. polyrhiza* genome (lines 7498 and 9509) (Fig. 3b). As expected given the loss of *CMT2*, there is no enrichment of CWA methylation in either genotype (Fig. 3c). There is a low level of CWG methylation in both lines, though (Fig. 3c). CWG methylation is dependent on *CMT3* (Gouil and Baulcombe 2016), which is present and expressed (Fig. 2c). Across the global range of *A. thaliana,* there is extensive variation at the *CMT2* locus including a nonfunctional *cmt2* allele that is associated with reduced genome-wide CHH methylation, but also the benefit of increased tolerance to heat stress (Shen *et al*. 2014; Dubin *et al*. 2015). Given that *S. polyrhiza* is globally distributed and thrives in a variety of climates and stresses, increased genotyping and phenotyping of diverse populations may reveal similar patterns of methylation-sensitive phenotypes. Intriguingly, *CMT2* is missing in the maize genome (Zemach *et al*. 2013), but also missing from the aquatic seagrass *Zostera marina* genome assemblies and annotations (Fig. 3b), suggesting that *CMT2* loss may be a shared feature that has evolved in multiple aquatic plants in the Alismatales order. Despite a lack of expression of key RdDM genes and sRNAs that normally function to target repetitive DNA, there has not been a recent detectable expansion of LTR retrotransposons in the *S. polyrhiza* genomes (Fig. 3d) (Wang *et al*. 2014; Michael *et al*. 2017), nor were they methylated in the typical CHH context (Fig. 1d). Specifically, only 3/1,114 (0.003%) and 6/1,510 (0.004%) LTR retrotransposons were enriched for CHH methylation in 7498 and 9509 genomes, respectively, and likely false positives (Zemach *et al*. 2013).

### Pericentromeric heterochromatin is present

**Fig. 4.**
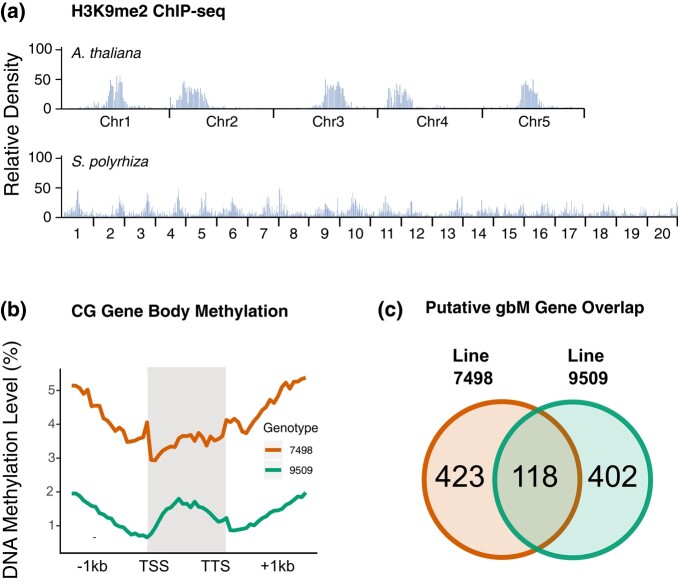
a) Distribution of H3K9me2 ChIP-seq peaks in *A. thaliana* and *S. polyrhiza* line 9509. b) Weighted gene body methylation plotted along coding sequence (CDS) regions spanning from TSS to TTS, plus or minus 1 kilobase. c) The overlap of blindly calling putative gene body methylated genes in 2 genotypes of *S. polyrhiza*.

The loss of CHH methylation, 24 nt het-siRNAs, and CMT2 suggests that the abundance of heterochromatin may also be low. Cao *et al*. (Cao *et al*. 2015) made an observation using 5mC and histone 3 lysine 9 dimethylated (H3K9me2) immunostaining, a common histone modification in heterochromatic regions of the genome (Bernatavichute *et al*. 2008), that *S. polyrhiza* and 4 other genera of the Lemnaceae lack strong signals of concentrated heterochromatic blocks of DNA. H3K9 methylation mediates CHG and CHH methylation through the action of CMT3 and CMT2, respectively (Stroud *et al*. 2014). To test if the CMT2 loss and the weak levels of CHG methylation are tied to a reduction of H3K9 methylation in *S. polyrhiza*, we performed ChIP-seq of H3K9me2 (Supplementary Table 3). H3K9me2 is sparsely distributed throughout the euchromatic chromosome arms and shows a discrete enrichment of a large domain within each chromosome (Fig. 4a). These relatively larger domains of H3K9me2 are ∼400–600 kb and presumably reflect the pericentromeric regions similar to observations in other angiosperms like *A. thaliana* (Fig. 4a). H3K9me2 occupies ∼15% of the line 9509 genome. In the 9509 genome, 746/1,510 (49.40%) of LTR retrotransposon annotations overlap H3K9me2 (Fisher's Exact Test, *P* < 0.001; Supplementary Table 4, Supplementary Fig. 4). Overall, H3K9me2 and heterochromatin appear normal in *S. polyrhiza*, especially when considering the small genome size split into 20 chromosomes.

### Gene body methylation is absent

Maintenance of DNA methylation at heterochromatin is associated with the establishment and maintenance of gene body DNA methylation (gbM) (Inagaki and Kakutani 2012; Bewick *et al*. 2016; Wendte *et al*. 2019; Zhang *et al*. 2020). It is characterized by an enrichment of CG DNA methylation between the transcription start site (TSS) and the transcription termination site (TTS) of genes (Tran *et al*. 2005; Bewick *et al*. 2017). Genes with gbM are typically moderately expressed throughout all tissues, long, and exhibit low rates of nucleotide substitutions compared to non-gbM genes (Takuno *et al*. 2016, 2017). Previously, Michael *et al*. described gene body methylation in *S. polyrhiza* line 9509 as “low” (Michael *et al*. 2017). Following this intriguing observation, we blindly quantified CG methylation in coding regions of each gene, only accounting for the number of methylated CG sites, total CG sites, and read coverage (Fig. 4c). In *S. polyrhiza*, this resulted in 541 and 520 putative gbM genes in lines 7498 and 9509, respectively, or 2–3% of the total gene annotation set. Comparing the 2 putative gbM gene sets, 118 genes overlap between the 2 genomes (blastp 1e-40), which is unexpected as gbM genes are often highly conserved (Takuno *et al*. 2016; Zhang *et al*. 2020) (Fig. 4b). These results are similar to another species that has lost gbM, *Eutrema salsugineum*, where roughly 500 genes were bioinformatically detected as having gbM signatures using a similar methodology (Bewick *et al*. 2016). This result is likely driven by a similar false positive rate of gbM gene detection in both species, as well as transposon misannotation, and that like *E. salsugineum,* gbM has been lost in *S. polyrhiza*.

The faithful establishment and maintenance of gbM is tied to a self-reinforcing feedback loop that relies on the interplay between CMT3 and H3K9me2 (Du *et al*. 2012; Stoddard *et al*. 2019; Wendte *et al*. 2019; Zhang *et al*. 2020). This is further supported by studies in *A. thaliana* whereby mutants that result in a loss of maintenance of heterochromatin lead to ectopic activity of CMT3 in gbM genes (Saze *et al*. 2008; Miura *et al*. 2009; Ito *et al*. 2015). In *S. polyrhiza*, we show that the CMT3/H3K9me2 feedback loop is weak in comparison to other angiosperms, even though H3K9me2 has a typical distribution throughout the genome. Therefore, it is possible that CMT3 activity is impaired, which leads to a weakly functioning feedback loop in *S. polyrhiza* and the loss of gbM. These results are consistent with proposed models from Wendte *et al*. (Wendte *et al*. 2019) and Inigaki and Kakutani (Inagaki and Kakutani 2012), in which CMT3 and H3K9me2 work coordinately to establish de novo gbM.

## Discussion

DNA methylation is a widespread chromatin modification that is typically found in all plant species (Ritter and Niederhuth 2021). By examining 1 aquatic duckweed species, *Spirodela polyrhiza,* we find that it has lost highly conserved genes involved in the methylation of DNA at sites often associated with repetitive DNA, and within genes, however, DNA methylation and heterochromatin are maintained during cell division at other sites. Consequently, small RNAs that normally guide methylation to silence repetitive DNA like retrotransposons are diminished. Despite the loss of a highly conserved methylation pathway, and the reduction of small RNAs that normally target repetitive DNA, transposons have not proliferated in the genome, perhaps due in part to the rapid, clonal growth lifestyle of duckweeds.

Ecological life history and developmental traits may strongly influence genome-wide patterns of DNA methylation and inheritance, especially relating to the suppression of transposon expansion over time. *S. polyrhiza* primarily reproduces via rapid clonal propagation rather than by flower production and sex (Ziegler *et al*. 2015), though some low-frequency instances of flowering have been reported (Khurana and Maheshwari 1980; Landolt and Kandeler 1987). The methylomes of other clonally propagated species (*Eucalyptus grandis*, *Fragaria vesca*, *Manihot esculenta*, *Theobroma cacao*, and *Vitis vinifera*) possess mCHH, although the levels are lower than nonclonally reproducing angiosperms (McKey *et al*. 2010; Niederhuth *et al*. 2016). This suggests that CHH reinforcement is linked to sexual reproduction, but is not necessary for transposon silencing as clonally propagated species rely more on maintenance DNA methylation. Resequencing of globally distributed *S. polyrhiza* accessions reveals very little per-site genetic diversity within the species, a low recombination rate, and weak purifying selection, but still a large effective population size (*N_e_*) (Ho *et al*. 2019; Xu *et al*. 2019). Few transposons exist in the ∼150 Mb *S. polyrhiza* genome, but a high ratio of solo-LTRs to intact LTR retrotransposons (Michael *et al*. 2017) suggests that LTR excision is actively occurring despite weak purifying selection. Individuals with fewer transposons and the ability to excise them may have selective advantages in large populations (Dolgin and Charlesworth 2006), given that a deleterious transposon insertion is unlikely to propagate and fix in a large clonal population, possibly compensating for the lack of a canonical RdDM pathway.

Several alternative hypotheses could explain the lack of CHH methylation and 24 nt het-siRNAs. DNA methylation and sRNA production may be limited to cell or tissue-specific regions, such as the developing meristematic region where daughter plantlets emerge. Tissue sampling for duckweeds is often performed on several individual plantlets combined, given the small size, which would reduce the detectable signal of tissue- or cell-type-specific changes and require more precise excision or cell-sorting techniques. It is also possible that these mechanisms may be active in flower tissue, which was not sampled in this study. Overall, *S. polyrhiza* displays a loss of CHH-type DNA methylation and heterochromatic siRNAs, which may be tied to its rapid asexual reproduction. Our work in *S. polyrhiza* demonstrates that reproductive success through rapid clonal propagation may benefit from the sacrifice of the RdDM and CMT2 pathways.

## Supplementary Material

jkae004_Supplementary_Data

## Data Availability

All raw small RNA, DNA methylation, and H3K9me2 ChIP-seq data are available at BioProject GSE161234. Epigenome browsers are available for *S. polyrhiza* line 7498 (http://epigenome.genetics.uga.edu/SchmitzLab-JBrowse/?data=spi_pol_7498) and for line 9509 (http://epigenome.genetics.uga.edu/SchmitzLab-JBrowse/?data=spi_pol_9509). Supplemental material available at G3 online.
