# Supplementary material for: The unusual predominance of maintenance DNA methylation in Spirodela polyrhiza
Source: G3 (Bethesda). 2024 Jan 8;14(4):jkae004. doi: 10.1093/g3journal/jkae004 (PMC10989885; doi:10.1093/g3journal/jkae004)
Supplement: jkae004_Supplementary_Data [file jkae004_supplementary_data.pdf]

Supplemental Figure 1. *DCL3* is not expressed under various stress conditions using aligned RNA-seq data of several stresses, including copper, kinetin, nitrate, and sucrose additions.

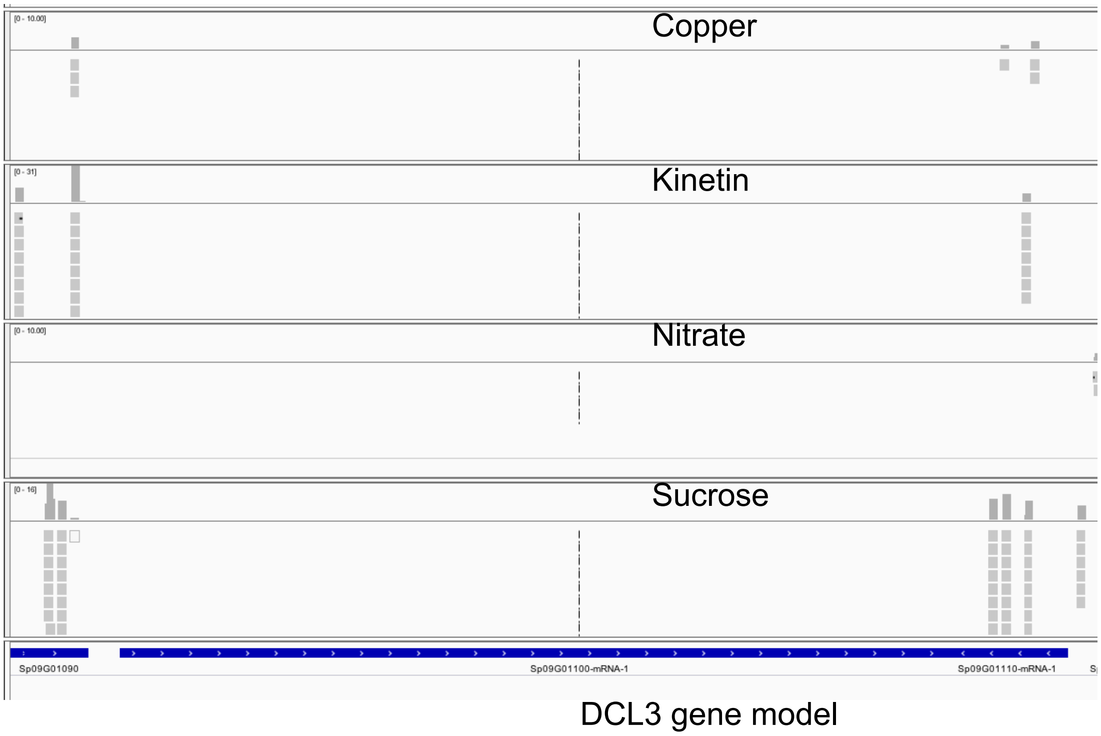

Supplemental Figure 2. Structure of the DCL3 gene. A) DCL3 protein structure with canonical Dicer-like PFAM domains. DEAD/DEADH-box helicase, Helicase C-terminal domain, Dicer dimer, PAZ, and two tandem Ribonuclease III domains are intact in the gene model when the protein was screened with hmmscan against the PFAM database. B) The *DCL3* gene model is not expressed when measured with RNA-seq read coverage, and it has a short promoter region (highlighted in red) that is possibly interrupted by the upstream U-box protein SP09G01090.

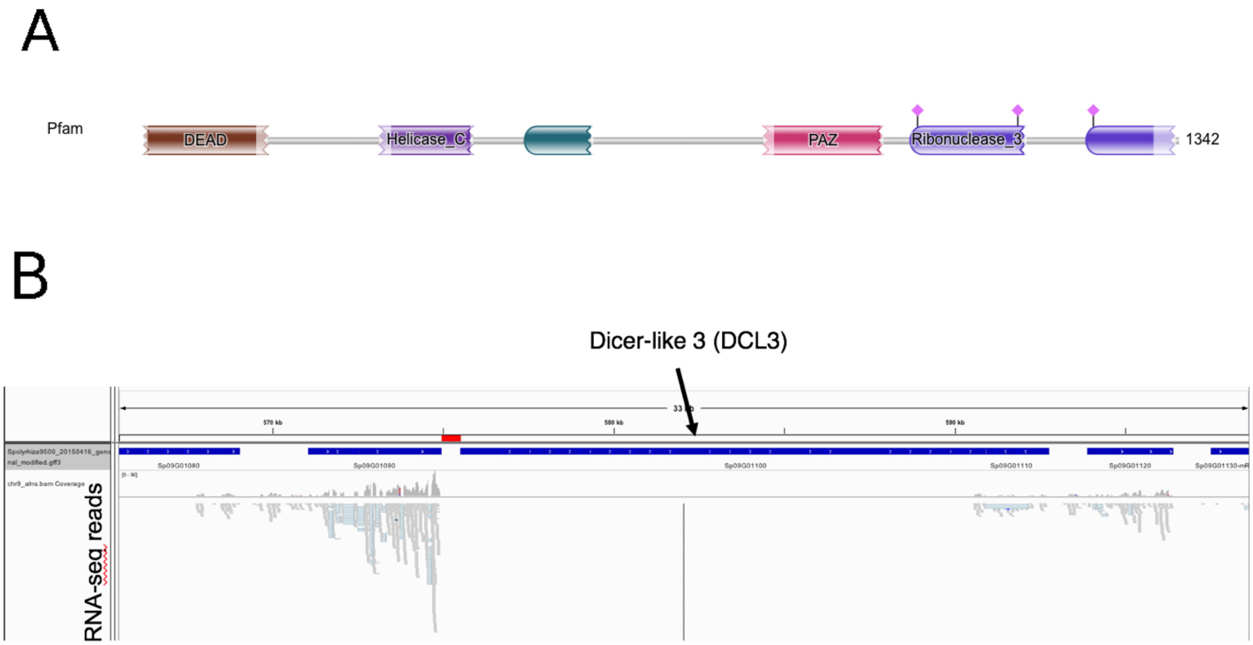

Supplemental Figure 3. A) Dicer-like protein family phylogenetic tree (DCL1, DCL2, DCL3, DCL4, DCL5) and B) ZMET/CMT Bayesian phylogenetic tree built using BEAST. Nodes are annotated with a ball size proportional to a posterior probability (see the key in each panel).

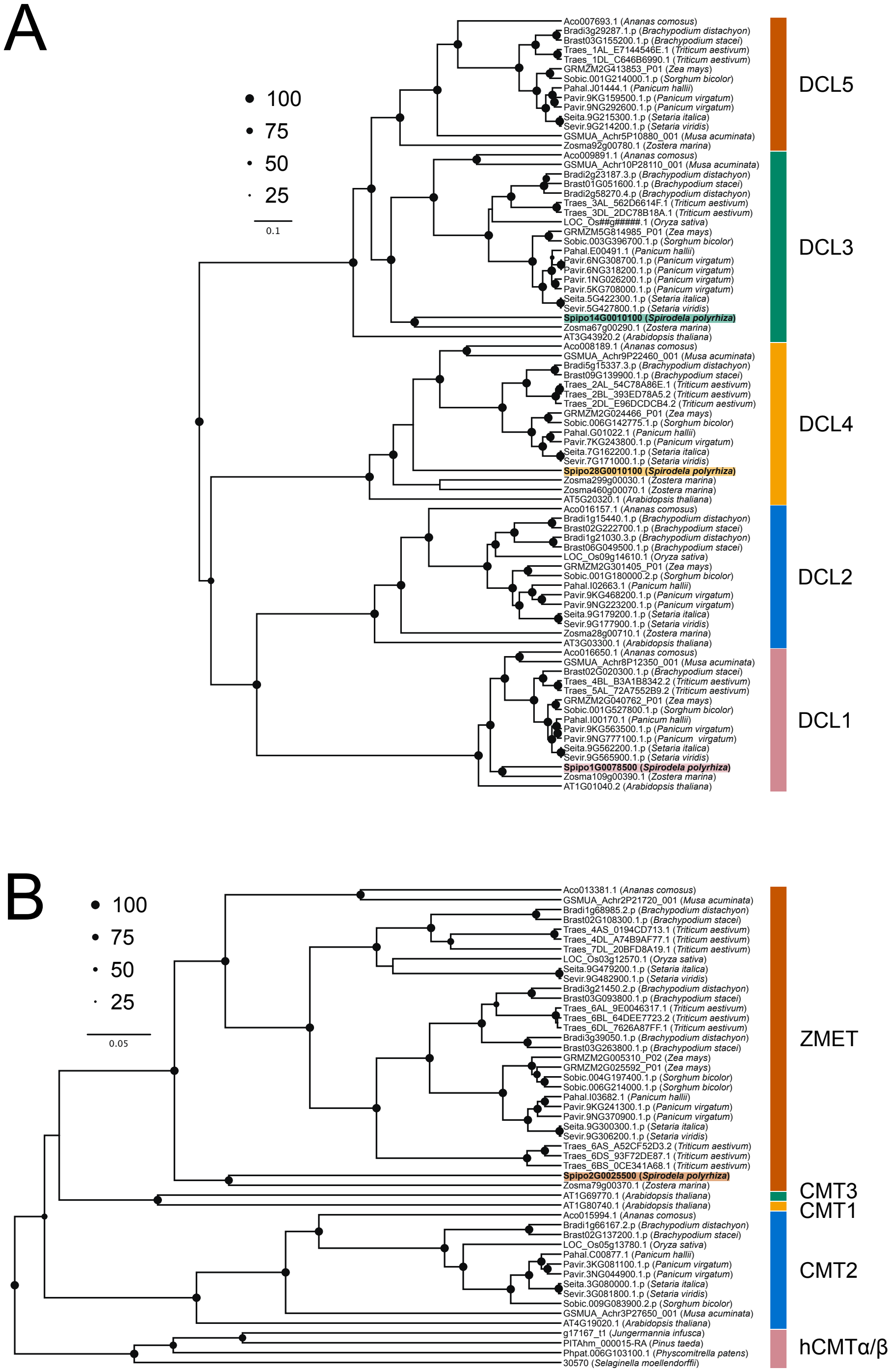

Supplemental Figure 4: Enrichment of H3K9me2 at LTR retrotransposons

H3K9me2 enrichment at LTR retrotransposons (Sp9509 Chromosome 1)

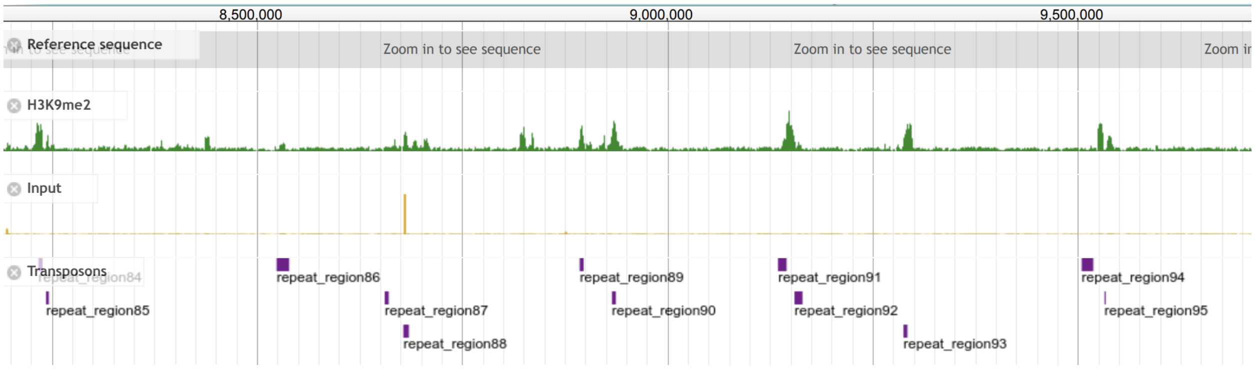

Supplemental Table 1. Sequencing and alignment statistics for *S. polyrhiza* line 7498 and 9509 MethylC-seq

Library	Raw Reads	Alignments to genome after PCR duplicate removal	Non-Conversion rate (%)
Sp9509_methylC	113,081,904	46,730,451	1.65
Sp7498_methylC	58,068,357	32,028,794	0.13

Supplemental Table 2. Sequencing and alignment statistics for *S. polyrhiza* line 7498 and 9509 small RNA data.

Library	Raw Reads	Adapter and 18-30 nt length trimmed reads	Perfect alignments to genome
7498_rep1	64,619,776	46,257,454	23,346,108
7498_rep2	67,122,721	51,974,074	24,270,971
9509_rep1	74,825,605	66,368,896	55,685,485
9509_rep2	73,778,606	66,612,352	48,810,499

Supplemental Table 3. Sequencing and alignment statistics for *S. polyrhiza* line 7498 and 9509 H3K9me2 ChIP-Seq

Library	Raw Reads	Alignments to genome after PCR duplicate removal
Sp7498_H3K9me2	30,509,305	26,286,244
Sp7498_input	45,911,383	37,887,965
Sp9509_H3K9me2	24,523,030	23,607,285
Sp9509_input	82,794,333	60,816,027

Supplemental Table 4. Fisher's Exact Test overlap of LTR retrotransposons and H3K9me2 peaks

	Overlapping an LTR	Not overlapping an LTR
Overlapping an H3K9me2 peak	713	508
Not overlapping an H3K9me2 peak	1,756	

*Two-tailed Fisher's Exact P-value = 2.0654e-184
